# mTOR Inhibition Elicits a Dramatic Response in PI3K-Dependent Colon Cancers

**DOI:** 10.1371/journal.pone.0060709

**Published:** 2013-04-09

**Authors:** Dustin A. Deming, Alyssa A. Leystra, Mohammed Farhoud, Laura Nettekoven, Linda Clipson, Dawn Albrecht, Mary Kay Washington, Ruth Sullivan, Jamey P. Weichert, Richard B. Halberg

**Affiliations:** 1 Division of Hematology and Oncology, Department of Medicine, University of Wisconsin, Madison, Wisconsin, United States of America; 2 Department of Oncology, University of Wisconsin, Madison, Wisconsin, United States of America; 3 Comprehensive Cancer Center Small Animal Imaging Facility, University of Wisconsin, Madison, Wisconsin, United States of America; 4 Division of Gastroenterology and Hepatology, Department of Medicine, University of Wisconsin, Madison, Wisconsin, United States of America; 5 Department of Pathology and Vanderbilt-Ingram Cancer Center, Vanderbilt University School of Medicine, Nashville, Tennessee, United States of America; 6 Research Animal Resources Center, University of Wisconsin, Madison, Wisconsin, United States of America; 7 Department of Radiology, University of Wisconsin, Madison, Wisconsin, United States of America; The University of Kansas Medical Center, United States of America

## Abstract

The phosphatidylinositide-3-kinase (PI3K) signaling pathway is critical for multiple cellular functions including metabolism, proliferation, angiogenesis, and apoptosis, and is the most commonly altered pathway in human cancers. Recently, we developed a novel mouse model of colon cancer in which tumors are initiated by a dominant active PI3K (FC PIK3ca*). The cancers in these mice are moderately differentiated invasive mucinous adenocarcinomas of the proximal colon that develop by 50 days of age. Interestingly, these cancers form without a benign intermediary or aberrant WNT signaling, indicating a non-canonical mechanism of tumorigenesis. Since these tumors are dependent upon the PI3K pathway, we investigated the potential for tumor response by the targeting of this pathway with rapamycin, an mTOR inhibitor. A cohort of FC PIK3ca* mice were treated with rapamycin at a dose of 6 mg/kg/day or placebo for 14 days. FDG dual hybrid PET/CT imaging demonstrated a dramatic tumor response in the rapamycin arm and this was confirmed on necropsy. The tumor tissue remaining after treatment with rapamycin demonstrated increased pERK1/2 or persistent phosphorylated ribosomal protein S6 (pS6), indicating potential resistance mechanisms. This unique model will further our understanding of human disease and facilitate the development of therapeutics through pharmacologic screening and biomarker identification.

## Introduction

The development of targeted therapies for the treatment of cancer has been a subject of great interest and effort. Until recently, new directed therapies have been studied in largely unselected populations. Limited efficacy has been demonstrated using this approach. However, as we evolve closer to an era of personalized medicine, each histological subtype of cancer is becoming better understood as a collection of rare cancers with each defined by its mutation profile. Therefore, the testing of targeted agents should be performed with a selected population carrying mutations known to activate the signaling pathways being targeted.

Human colonic tumors contain several possible oncogenic driver mutations which could potentially be targeted, including *KRAS*, *BRAF*, and *PIK3CA* [1] These mutant kinases have been key targets for the continued development of therapeutic agents. They have also been important in understanding the biology of resistance to the epidermal growth factor receptor directed therapies, cetuximab and panitumumab [2], [3]. Despite significant advances in the treatment of this deadly disease, colorectal cancer remains the second leading cause of cancer related death in the United States [4]. To advance the treatment options for patients, further investigation regarding the biology of these mutations is necessary. This will provide an improved understanding of which patients are most likely to respond to particular therapies.

*PIK3CA* mutations occur in 20 to 30% of human colorectal cancers [5], [6]. Three hotspot mutations are commonly found, including H1047R, E542K, and E545K, which result in a constitutively active form of the PI3K p110α catalytic subunit [7]. This dominant active PI3K then results in increased AKT/mTOR pathway signaling and increased cellular proliferation (Figure S1) [8]. While several investigators have examined the effects of these mutations in cell lines, our laboratory recently developed a murine model of colon cancer that is initiated by a dominant active PI3K (FC PIK3ca*) [9]. In this model, large moderately differentiated mucinous invasive adenocarcinomas develop in the proximal colon by only 50 days of age. These tumors are initiated by a non-canonical pathway independent of aberrant WNT signaling. Since these tumors are initiated by activated PI3K, we aimed to determine if these tumors are dependent on this pathway. Here, we demonstrate that the treatment of FC PIK3ca* mice with the mTOR inhibitor, rapamycin, results in a dramatic response in advanced colon cancers. This indicates that human tumors dependent on the PI3K/AKT pathway are likely to be susceptible to inhibitors of downstream mediators.

## Materials and Methods

### Mouse Husbandry

All animal studies were conducted under protocols approved by the Institutional Animal Care and Use Committee at the University of Wisconsin-Madison, following the guidelines of the American Association for the Assessment and Accreditation of Laboratory Animal Care. Homozygous *FC^+^* female mice (FVB/N-Tg(Fabp1-Cre)1Jig; NCI Mouse Repository; Strain number - 01XD8) were crossed to homozygous PIK3ca* ^+^ male mice (C57BL/6-Gt(ROSA)26Sor^tm7(Pik3ca*,EGFP)Rsky/^ ^J^; The Jackson Laboratory; Stock Number - 012343) to generate FC PIK3ca* mice used in this study. Mice were genotyped for *FC* and PIK3ca* as described previously [10], [11].

### Animal Treatment

FC PIK3ca* mice between 50 and 60 days of age were selected for study enrollment as long as they were not moribund. Baseline dual hybrid ^18^F-FDG PET/CT scans were performed prior to and 15 days following treatment initiation. The tumor size was subjectively determined from the pre-treatment raw images and used for stratification during randomization to the rapamycin and control arms. Animals in the control arm received ethanol dissolved in water to a 1% final concentration by oral gavage daily for 14 days. Animals randomized to the rapamycin arm received 6 mg/kg of rapamycin (LC Labs, Woburn, MA) by oral gavage per day for 14 consecutive days. Rapamycin was dissolved in ethanol to a concentration of 50 mg per ml prior to suspending in water to a final volume of 200 µl for administration.

### Imaging

Animals were fasted for at least 6 hours prior to injection of ^18^F-FDG (160 µCi; IBA Molecular, Romeoville, IL) or ^18^F-FLT (140 µCi; University of Wisconsin Cyclotron, Madison WI). After injection, the animals were kept under anesthesia for 60 minutes and then prepared for virtual colonography as described previously [12]. A 10-minute PET acquisition was performed, followed immediately by CT scanning. Maximum intensity projections were created in Siemens Inveon Research Workplace (Knoxville, TN). The PET images were reconstructed using OSEM3D/MAP (OSEM3D, 2 iterations; MAP 18, iterations 16 subsets). Attenuation correction was performed using the CT data. The CT images were reconstructed using standard conebeam reconstruction. Baseline and post-treatment PET scans were normalized to injected dose, dose decay, activity, and weight. Tumor volumes were estimated from measurements on the PET/CT scans (Figure S2). PET imaging was utilized to locate tumors prior to volume estimation. Tumor volumes can only be estimated, as delineating the exact tumor boundaries is difficult. This is because these cancers are not luminal and subtle FDG signal changes related to the hyperplastic normal epithelium surrounding the tumors exist. Tumor volumes in each cohort were compared using a two-sided Student’s exact t-test. A p value of less than 0.05 was considered statistically significant.

### Histology and Immunohistochemistry

Mice were euthanized by CO_2_ asphyxiation following their post-treatment imaging. The small bowel and colon were removed, flushed with PBS, split lengthwise, splayed out, and fixed in 10% buffered formalin for 24 hours. Tissues were then stored in 70% ethanol, processed, embedded in paraffin, and cut into 5 µm sections. Every tenth section was stained with hematoxylin and eosin (H&E) for histological review. Immunohistochemistry was performed using the Histomouse™ Max Broad Spectrum (DAB) kit as instructed by the manufacturer (Invitrogen, Carlsbad, CA) and as previously described [9]. Immunofluorescence was performed using a similar protocol with the following changes: 5% milk in PBS was used to block tissues. All steps after incubation with the primary antibody and washing were omitted and replaced with the following: incubated with AlexaFluor 488 goat anti-rabbit IgG fluorescent secondary antibody (1∶1000, Invitrogen) for one hour, washed in PBS, and mounted using the ProlongGold Antifade Reagent with DAPI (Invitrogen). The primary antibodies included: anti-pAKT (Ser473, 1∶100, Cell Signaling Technology, Beverly, MA), anti-pS6 (1∶200, Cell Signaling Technology), anti-Ki67 (1∶1000, Cell Signaling Technology) and mouse anti-β-catenin (1∶200, BD Biosciences - Clone 14, San Diego, CA).

### Western Blot Analysis

Tissue samples were excised and flash frozen. After 24 hours, the samples were sonicated in T-PER tissue protein extraction reagent (Thermo Scientific, Pittsburg, PA), proteasome inhibitor cocktail (Sigma-Aldrich, St. Louis, MO), and phenylmethylsulfonyl fluoride (PMSF, Sigma-Aldrich). Extracted protein was then run as previously described [9]. Primary antibodies against p110α, pAKT (Ser473), AKT (pan, 11E7), total Tuberin/TSC2 (D93F12), pmTOR (Ser2448), pS6K1 (p70 S6 kinase, Thr389), pS6 (Ser235/236), Total S6 (5G10), cleaved caspase 3, pERK1/2 (Thr202/Tyr 204) and total ERK 1/2 (Cell Signaling Technology) were incubated in bovine serum albumin (Sigma-Aldrich) at a 1∶1000 ratio for 16 hours. Anti-GAPDH antibody (Cell Signaling) was utilized as a loading control at a ratio of 1∶5000.

## Results

### FC PIK3ca* Mice Develop Proximal Colon Cancers that can be Followed Longitudinally for Treatment Studies

FC PIK3ca* mice rapidly develop moderately invasive mucinous adenocarcinomas [9]. Importantly for this study, the tumors in these mice can be detected by dual hybrid ^18^F-FDG or ^18^ F-FLT PET/CT colonography (Figure S3 *A* and *B*, respectively). In our recent description of this model, we evaluated the development of tumors in the colon over time [9]. Invasive adenocarcinomas were identified in 75% of mice at just 40 days of age. The vast majority of mice become moribund by just 60 to 80 days of age. Given the goal of this study to measure the effect of rapamycin on pre-existing colon cancers with a dominantly active PI3K, we placed into our therapeutic study 22 FC PIK3ca* mice at 55 days of age, an age when most have pre-existing cancer, but have not yet become moribund. The mice were stratified into groups based on gender and pretreatment tumor size as estimated from baseline dual hybrid ^18^F-FDG PET/CT colonography. A volume of 50 mm^3^ was used as a cut-off to determine large versus small tumors. These mice were then randomized into two treatment arms, receiving either placebo or rapamycin by oral gavage. Baseline characteristics are displayed in Table 1.

**Table 1:** FC PIK3ca* mice baseline characteristics and tolerability of rapamycin versus placebo.

			Pre-Tx tumors, N by size		Mouse weight, mean (g)			
Treatment arm	Sex	N	Large (>50 mm^3^)	Small (<50 mm^3^)	Pre-Tx	Post-Tx	Deaths on study	Received all doses
Placebo	Male	6	4	2	24.9	23.7	2	4/6
	Female	5	1	4	20.1	21.3	0	5/5
Rapamycin	Male	7	2	5	24.8	23.9	0	7/7
	Female	4	2	2	19.2	20.1	0	4/4

Twenty-two mice at 55 days of age were randomized 1∶1 to rapamycin or placebo treatment arms. Mice were stratified based on gender and pretreatment tumor size. These mice tolerated the treatment well as demonstrated by minimal change in weight from baseline. In the placebo cohort, two mice became moribund prior to study completion. In both instances, large proximal colon tumors caused obstructive enteropathy. All mice in the rapamycin treated cohort completed the treatment course as intended. Tx, treatment.

### FCPIK3ca* Mice Tolerate Rapamycin Treatment

Rapamycin was administered to FC PIK3ca* mice at a dose of 6 mg/kg/day by oral gavage for a total of 14 consecutive days, which had been shown previously to be tolerable to mice [13]. The FC PIK3ca* mice also tolerated this treatment well (Table 1). No significant change in activity level or weight was noted between the placebo and treatment cohorts throughout the study period. Two mice in the placebo arm became moribund due to colonic obstruction from large proximal colon tumors and were sacrificed prior to completion of the intended treatment course. Both of these mice had large tumors on baseline imaging with volumes over 80 mm^3^.

### Rapamycin Induces a Significant Tumor Response in FC PIK3ca* Mice

**Figure 1:**
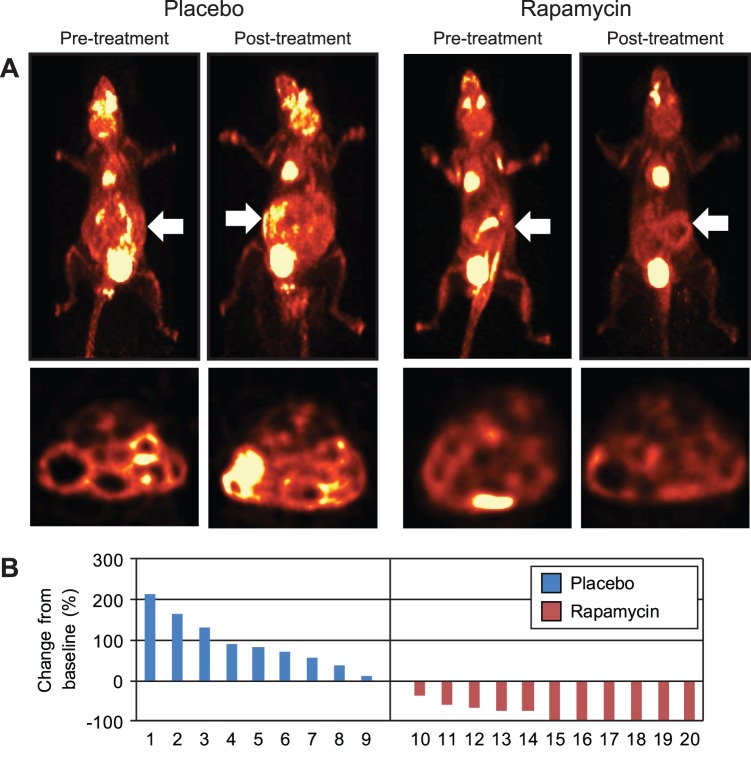
Longitudinal monitoring with dual hybrid ^18^F-FDG PET/CT colonography revealed a dramatic tumor response in FC PIK3ca* mice treated with rapamycin. A group of 22 FC PIK3ca* mice were imaged with dual hybrid ^18^F-FDG PET/CT colonography and stratified based on tumor size. Mice were then treated with a placebo (ethanol dissolved in drinking water) or rapamycin 6 mg/kg/day by oral gavage for 14 days. Following completion of the treatment course, PET/CT colonography was repeated to evaluate for tumor response. In the mice receiving placebo, the tumors increased in size during the 14 day treatment period (*A, left*). In the rapamycin arm a significant response was noted (*A, right*). Projection and axial views are presented on the top and bottoms, respectively. The tumors were located on PET images and volumes were measured from CT scans data. The percent change in tumor volume for each tumor is displayed in a waterfall plot (*B*).

After 14 days of treatment, the mice in both the placebo and rapamycin arms were imaged a second time to assess treatment efficacy. After normalization of the imaging data, a dramatic response was noted in the rapamycin-treated mice as compared to controls (Figure 1, Figure S4, and Table S1). In multiple animals, FDG activity consistent with tumor tissue could not be found following rapamycin treatment. The PET/CT images were used for tumor localization and the volumes were estimated based on measurements from these images (Figure S2). In the placebo arm, tumor volume nearly doubled in size from baseline with an increase from baseline of 96%. This dramatic change was expected as these cancers grow quite quickly in this model. In the rapamycin cohort, there was a marked reduction in tumor volume, with only 16.9% of the baseline mass still being present on average (Figure 1*B* and Table S1). Not all tumors responded to the same degree, albeit none of the cancers in the rapamycin arm were shown to increase in size while receiving treatment. Since these lesions are quite sensitive to inhibition of mTOR by rapamycin, this further indicates that they are dependent on the PI3K/AKT/mTOR signaling cascade.

**Figure 2:**
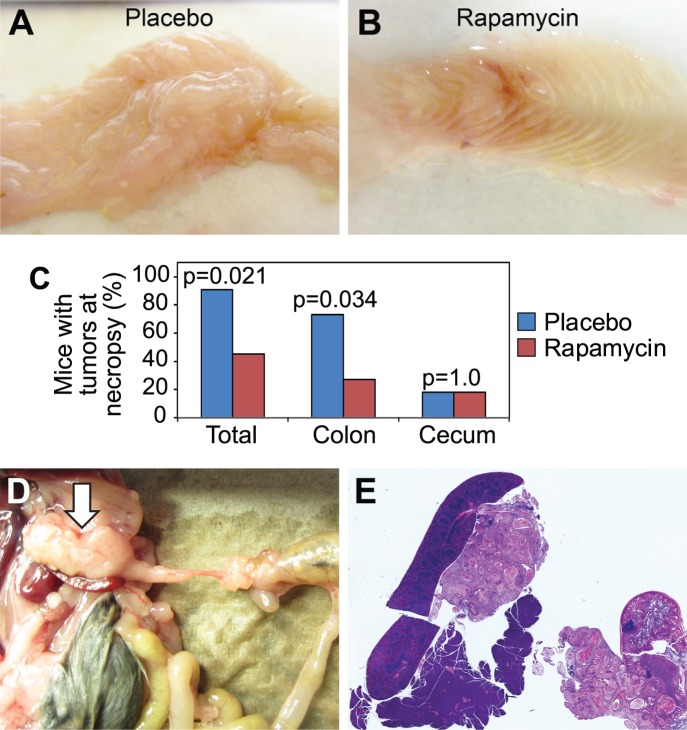
Tumor response in FC PIK3ca* mice was confirmed at necropsy and metastatic disease was identified in the control arm. Following post-treatment imaging, necropsy was performed. The colon was excised and split lengthwise. Large submucosal tumors were seen in mice treated with placebo (*A*). A dramatic response was observed at necropsy in the rapamycin-treated cohort (*B*). Hyperproliferation was diminished in the normal proximal colon and minimal residual tumor was identified. 10 of 11 FC PIK3ca* mice in the placebo cohort had identifiable tumors while only 5 of 11 mice in the rapamycin arm had identifiable tumors (*C*). One mouse in the placebo cohort was found to have metastatic disease from a cecal tumor (*D*). A large lesion within the mesentery was identified abutting the spleen and pancreas (*D, arrow*). On histological sectioning a similar morphology was noted between the metastatic deposit and the primary tumor within the cecum (*E*).

To confirm the response data acquired with the PET/CT colonography, necropsy was performed following imaging acquisition. A significant response was also seen at necropsy (Figure 2). Using an intent-to-treat analysis, cecal and/or colon tumors were grossly present in 10 of 11 mice in the placebo arm, but were present in only 5 of 11 mice in the rapamycin arm (Figure 2*C*, p = 0.021), indicating a significant treatment effect with single agent rapamycin therapy. In the placebo arm, 8 of 11 mice had colon tumors, while cancers were present in only 3 of 11 rapamycin-treated mice (Figure 2*C*, p = 0.034). Both groups had an equal number of cecal tumors, with 2 of 11 mice having them in each arm. One of the FC PIK3ca* mice in the control group with a cecal tumor also developed metastatic disease with the spread of cancer to the mesenteric tissue abutting the spleen and pancreas (Figure 2*D and E*). No evidence of metastatic disease was detected in any mice treated with rapamycin.

### FC PIK3ca* Tumors Demonstrate p110* Expression and Activation of the PI3K Pathway that is Decreased with Rapamycin Treatment Resulting in Tumor Response and Reduced Proliferation

**Figure 3:**
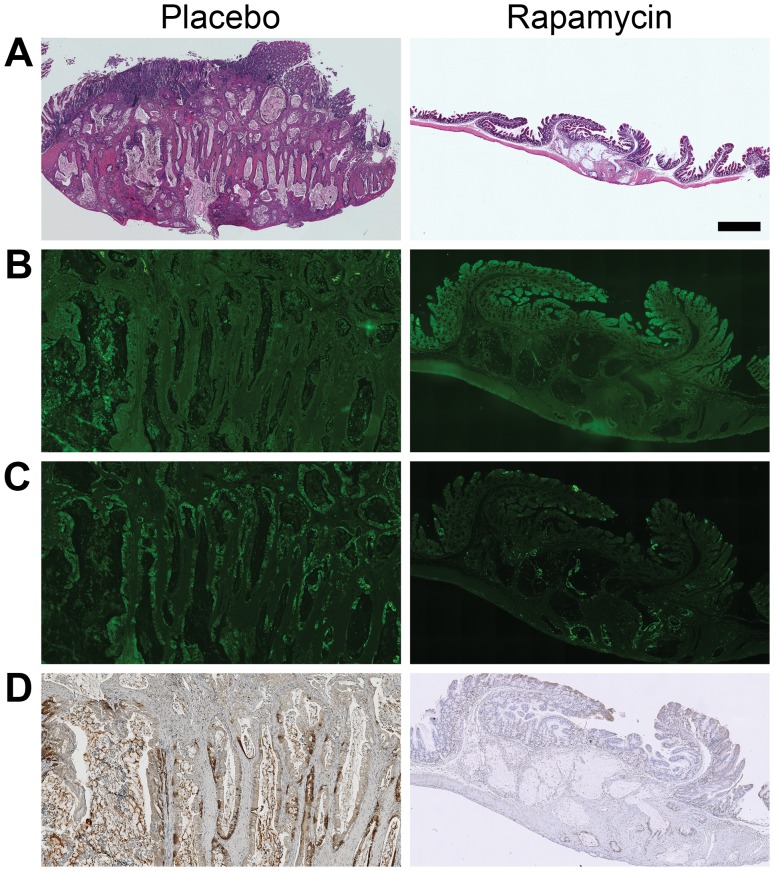
Staining of histological sections demonstrates tumor response, decreased PI3K signaling, and decreased cell proliferation following rapamycin treatment. Following necropsy, colonic tumor tissue with adjacent colon epithelial tissue was formalin-fixed, paraffin-embedded, and underwent histological sectioning. Tissue was stained with H&E (*A*). Compared to placebo, a dramatic decrease in size was noted in the tumors from rapamycin-treated mice. The tumor pictured in *A, rapamycin* is from a FC3K mouse treated in a pilot of this study and chosen for this figure due to the observed dramatic partial response. In addition, the malignant columnar epithelium surrounding the mucinous lakes was diminished following rapamycin treatment as compared to control. Fewer pAKT and pS6-positive cells were observed in the tumors from mice treated with rapamycin as compared to tumors from mice given placebo (*B* and *C,* respectively). Tumor proliferation, as measured by Ki67, was also reduced in tumors from mice treated with rapamycin compared to those treated with placebo (*D*). Size bar: *A*, 1 mm. *B–D* are ∼3× enlargements.

Following necropsy, the colon tumors were isolated and prepared for histological sectioning. H&E staining demonstrated hyperplastic epithelial tissue and moderately differentiated invasive mucinous adenocarcinomas as expected in the placebo cohort (Figure 3*A*, left). In the rapamycin-treated mice, a significant reduction in the hyperplasia of the epithelial tissue was noted, and the tumor size was reduced (Figure 3*A* *,* right). The columnar malignant cells typically encircling the mucinous lakes were gone in the majority of the tumors, resulting in fewer cells staining strongly for pAKT and pS6 in tumors from rapamycin-treated mice compared to controls (Figure 3*B and C* *,* respectively). Diminished cellular proliferation was noted following rapamycin treatment as demonstrated by decreased Ki67 staining (Figure 3*D*).

**Figure 4:**
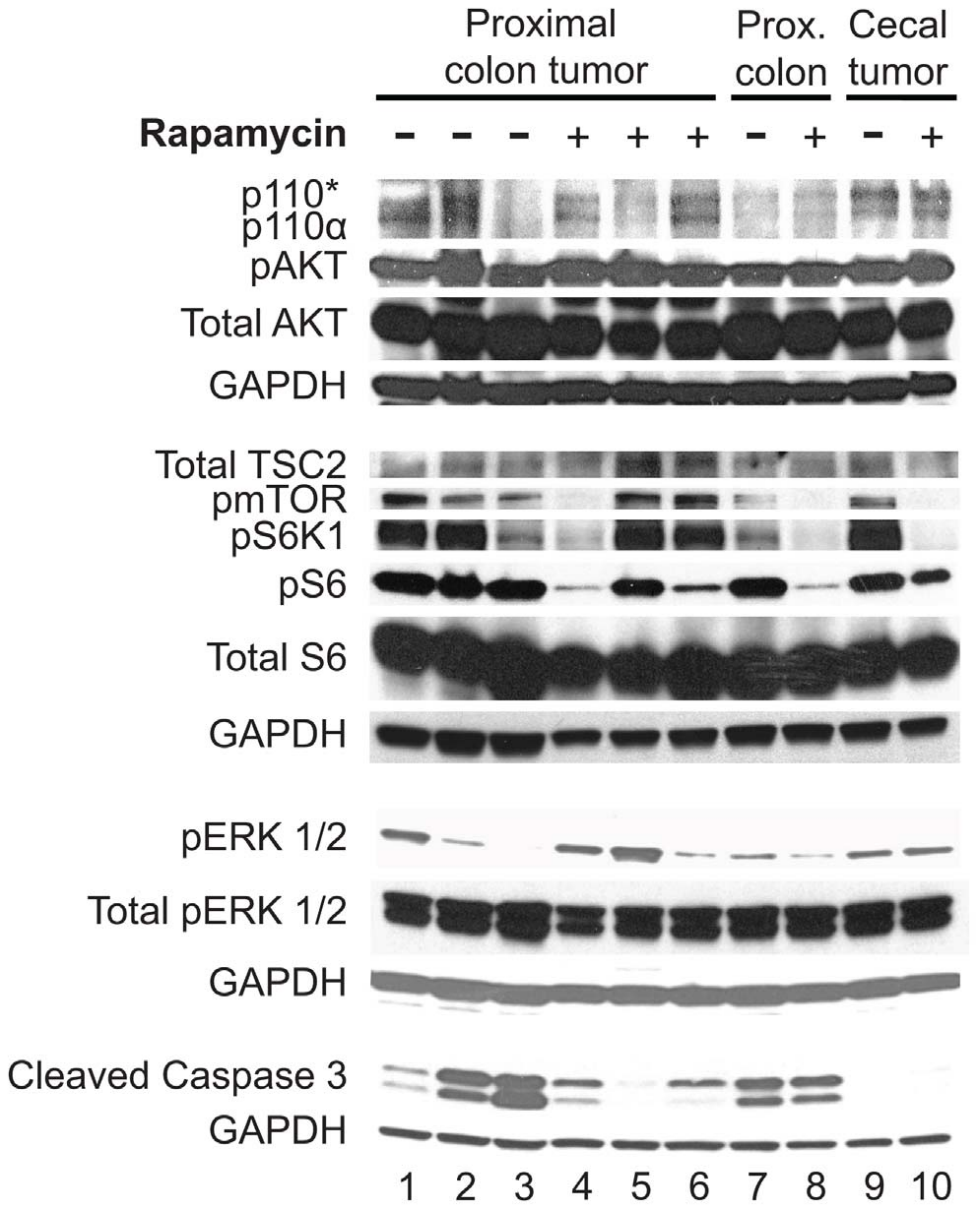
Upstream activation of the PI3K pathway was unperturbed, but downstream signaling varied with response to rapamycin treatment. All FC PIK3ca* tumors and hyperplastic colon tissue demonstrated expression of p110*, the dominant active catalytic subunit of PI3K in this model. This resulted in increased phosphorylation of AKT^9^. The levels of pAKT and total AKT were not altered in response to rapamycin treatment. TSC2 total levels also did not vary. The levels of pmTOR, pSK1, and pS6, however, varied with rapamycin treatment. In tumors from the placebo cohort (−), high levels of pS6 were observed. In tumors that were treated with rapamycin (+), decreased levels of pS6 were seen in the majority of tumors and correlated with increased response on PET imaging. Increased pERK1/2 was observed in some placebo-treated cancers and also the rapamycin-treated tumor with persistent pS6 signaling. FC PIK3ca* tumor and hyperplastic tissue had increased cleaved caspase 3 due to increased cell turnover. A reduction in cleaved caspase 3 was identified in tumors possessing increased pERK1/2 signaling indicating that tumors with increased pERK may be resistant to rapamycin treatment through decreased apoptosis. GAPDH was used as a loading control.

Following treatment with rapamycin, the upstream activation of the PI3K pathway remained unperturbed as expected (Figure 4). At necropsy, some tumor tissue was flash frozen prior to protein preparation and quantification. The protein p110*, the dominant active form of PI3K used in this model, was expressed in the tumors and normal epithelium of the proximal colon in all animals regardless of treatment. Significant pAKT expression was also noted in the tumors and normal proximal colon tissue. The level of pAKT does not appear to be affected by rapamycin treatment. Proteins pmTOR, pSK61, and pS6 were reduced in response to rapamycin treatment in most tissues, though some tumors did clearly demonstrate continued activation of these proteins. The tumors with persistent pS6 signaling had less of a response to rapamycin treatment as noted on PET imaging.

### Resistance to Rapamycin Treatment of FC PIK3ca* Mice Might be Mediated through pERK Up-regulation and Persistent pS6 Signaling Leading to Decreased Apoptosis

Some tumors had a limited response to rapamycin treatment. We hypothesized that this might be related to up-regulation of the Raf/MEK/ERK signaling cascade or persistent pS6 signaling. ERK1/2 up-regulation has previously been described in response to rapamycin treatment [14], [15]. Levels of pERK1/2 were evaluated in normal colon tissue, proximal colon tumors, and cecal tumors (Figure 4). pERK1/2 was found to be dramatically up-regulated in one mouse from each treatment arm. In a group of ten tumors from untreated FC PIK3ca* mice, six demonstrated some degree of pERK1/2, indicating possible mechanism(s) for intrinsic resistance (Figure S5 *A*). In addition, all tissues treated with rapamycin did display an increase in pERK1/2 over controls. These data indicate that a subpopulation of FC PIK3ca* tumors possess activation of ERK signaling. This signaling might be responsible for some degree of resistance to mTOR inhibition, since the ERK pathway is not inhibited with this treatment strategy. This resistance may be intrinsic to the tumor or induced secondary to the treatment.

To confirm that WNT signaling is not aberrant in these tumors, as described previously, [9] we performed histological analysis examining for evidence of nuclear β-catenin. No nuclear β-catenin was seen in control tumors (Figure S5 *B*). In addition, the tumor remnants from rapamycin-treated mice were also examined for aberrant WNT signaling to determine if induction of this pathway may be a further mechanism decreasing the sensitivity of these tumors to rapamycin. No evidence of nuclear β-catenin was demonstrated in the tumors of rapamycin-treated mice (Figure S5 *B*).

**Figure 5:**
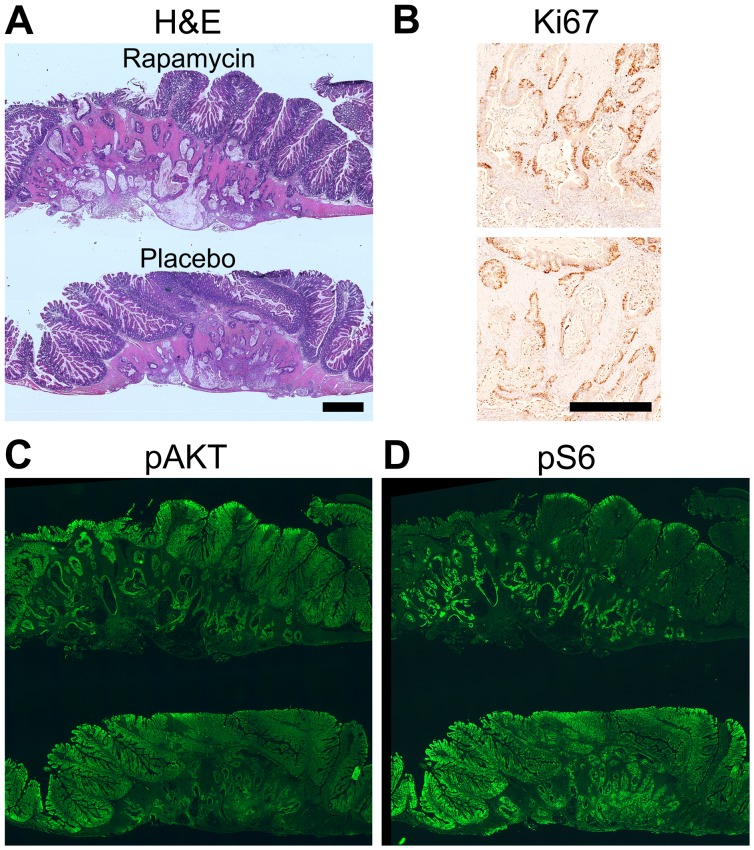
Persistent FC PIK3ca* tumors demonstrated a differential response in pS6 in the hyperplastic and tumor tissue following rapamycin treatment. A tumor resistant to rapamycin (top in each panel) and a tumor from a placebo-treated FC PIK3ca* mouse (bottom in each panel) were embedded in the same histological block. A similar phenotype and proliferation rate were noted on H&E and Ki67 staining, respectively (*A* and *B*). No significant difference in pAKT was seen between the tumors from the placebo and rapamycin treated mice (*C*). In the tissue from the placebo-treated mouse, increased pS6 was seen in the tumor tissue and overlying hyperplastic tissue as expected (*D*). Interestingly, in the tissue from the rapamycin-treated FC PIK3ca* mouse, decreased pS6 is noted in the hyperplastic tissue, but increased pS6 signaling remains in the resistant tumor tissue despite rapamycin treatment. This indicates that persistent pS6 setting within the tumors may be the mechanism of tumor resistance. This same staining pattern was observed in all tumors (3/3) with some neoplastic cells remaining after treatment with rapamycin that were examined. Size bars: *A*, *C* and *D*, 1 mm; *B*, 500 µm.

Histological examination of a representative rapamycin-resistant FC PIK3ca* tumor demonstrated a similar morphology to cancers from controls (Figure 5). In addition, no differences in cellular proliferation or levels of pAKT were noted (Figure 5*C*). In a tumor from a control mouse, pS6 was noted in the mucosal hyperplasia as well as the tumor tissue, as expected (Figure 5*D*, bottom). However, in the tumor from a rapamycin-treated mouse, a decrease in pS6 in the normal epithelial tissue was observed, but pS6 signal persisted in the remaining cancer tissue (Figure 5*D*, top, Figure S6 *A*). The amount of pS6 present in these resistant cancer cells exceeds what is seen in the responding cancer in Figure 3. This indicates a differential response in the normal tissue compared to the tumor tissue and might be a mechanism of resistance to mTOR inhibition in this setting.

## Discussion

Targeting oncogenic pathways has led to recent exciting advances in multiple cancers, including vemurafenib in *BRAF* mutant melanoma, erlotinib in *EGFR* mutant non-small cell lung cancer, and crizotinib in lung cancer with the EMLA4-ALK translocation [16]–[18]. These agents are used in the setting of specific genetic alterations encoding oncogenic proteins. Targeting these driver proteins results in a high response rate to agents directed against them. This approach allowed for the expedient evaluation and FDA approval of these agents for these indications [19]. The development of these agents in specific molecular settings has led to realization that each histological type of cancer is actually a collection of numerous rare subtypes. These subtypes are each differentiated by their profile of mutations.

The PI3K/AKT pathway is the most common genetically altered signalling cascade in cancer. Significant clinical interest in targeting the PI3K/AKT/mTOR pathway continues to increase as novel inhibitors of this pathway continue to be developed. Inhibition of the PI3K pathway in cancers possessing *PIK3CA* mutations has demonstrated clinical benefit in breast and gynecologic malignancies, but the presence of these mutations alone does not predict sensitivity to these approaches [20]. The subpopulation of patients who are most likely to benefit from the continued development of novel inhibitors of the PI3K pathway remains unclear.

Until recently, the effects of an activated PI3K on the mammalian intestine had not been investigated. We were the first to describe the development of advanced invasive mucinous adenocarcinomas developing in the proximal colon as a result of expression of a dominant active PI3K [9]. Interestingly, these tumors develop rapidly, without a significant luminal component or aberrations in WNT signaling, indicating a non-canonical mechanism of tumor initiation. Here, we demonstrate that the tumors forming in FC PIK3ca* mice are largely dependent upon the PI3K pathway. In this model, a dominant active PI3K is expressed in the colon resulting in tumor initiation. These cancers are very aggressive and are able to be followed longitudinally with dual hybrid ^18^F-FDG PET/CT colonography. After just two weeks of rapamycin therapy a significant response was observed with imaging and confirmed at necropsy. These results indicate that FC PIK3ca* tumors are dependent on this pathway. If similar tumors are present in humans, inhibition of the PI3K pathway could result in significant clinical benefit for appropriate patients.

We hypothesize that a subpopulation of human tumors form as a consequence of a dominant active PI3K and that these tumors are dependent upon this pathway. In a recent pathological series, activating mutations in *PIK3CA* were observed more commonly in mucinous colon cancers in humans, similar to our model, and were associated with worsened prognosis [21]. Activating mutations in *PIK3CA* were inversely associated with the translocation of β-catenin [21]. Translocation of β-catenin would be expected if these tumors were initiated by aberrant WNT signaling as part of the previously described canonical mechanisms of tumorigenesis in colon cancers [22]. Together, these observations indicate that a subgroup of human colon cancers arise in the setting of activated PI3K, similar to what is seen in our model. Only a small number of tumors have been interrogated for aberrations in WNT signaling and mutations in *PIK3CA*, thus additional investigations are warranted to further characterize this patient population. Based on these prior studies, we believe that this likely represents 1–5% of all colon cancers.

Even with the significant responses seen in this study, resistance to rapamycin therapy was identified. Increased pERK1/2, pS6, or both were observed in tumors persisting despite rapamycin treatment. These resistance mechanisms may be intrinsic to the tumors or induced secondary to the rapamycin treatment. Rapamycin is known to inhibit ribosomal S6 kinases (S6K1 and S6K2) through its interaction with mTOR [23]. The inhibition of the S6Ks diminishes downstream phosphorylation of S6 at Ser^240/244^ and Ser^235/236^ [24]. Studies in S6K1/S6K2-null mouse embryonic fibroblasts confirmed that these S6Ks were the major kinases affecting S6, but also demonstrated a MEK1/2 dependent mechanism of S6 phosphorylation at Ser^235/236^ [25]. In addition, ERK1/2 has been demonstrated to activate the p90 ribosomal S6 kinase (RSK), which can subsequently phosphorylate S6 at Ser^235/236^ independent of mTOR signaling (Figure S7) [26]. In all tumors from FC PIK3ca* mice, we observe phosphorylation of S6 thought to be related to activation of the PI3K pathway. In a subset of tumors, we also observe increased pERK1/2 and persistent pS6. These data indicate that the alternate phosphorylation of S6 mediated by ERK1/2 activation of RSK might be a mechanism by which these tumors are resistant to rapamycin. Combinations of targeted therapies will probably be necessary in some circumstances to overcome these resistance mechanisms. PI3K and Raf/MEK/ERK pathway inhibitor combination regimens continue to be an active area of preclinical and clinical research [27]–[29].

Though rapamycin is a well known inhibitor of mTOR and the PI3K pathway, new agents continue to be developed to target this pathway. Agents targeting PI3K, AKT, and mTORC1/mTORC2 are in clinical development. We hypothesize that these agents would have significant response rates in our model system [30]–[32]. It is likely that each of these agents would, however, have different side-effect profiles and mechanisms of resistance. BYL719 (Novartis) is the first alpha isomer specific PI3K inhibitor and may hold significant advantages over other agents with a hopefully improved safety profile compared to other pan inhibitors [33].

As we begin to expand our capabilities to personalize cancer treatments based on molecular features, this will necessitate subdivision of each histological type of cancer to collections of rare cancers defined by their mutation profiles. Acquiring genomic information as it relates to treatment response will be vital for the proper development of combinations of targeted therapies. Using murine models, we can control the mutation profile to quickly determine the best strategies to target these cancers. The findings can then be translated to the clinic, where patients can receive drugs dictated by the mutation profiles of their tumors. Preclinical testing of treatment strategies will be essential as the subtypes of each cancer become exceedingly rare and thus more difficult to study on a large scale in the clinic. *PIK3CA* mutations are a promising target in colorectal cancer, among others; now we need to optimize the administration of our therapies and identify the patient populations most likely to benefit.

## Supporting Information

Figure S1 **The PI3K signaling pathway mediates vital cellular functions.** The PI3K is commonly altered in human cancers and has been targeted for many new directed therapies.
(EPS)

Figure S2 **Volume estimation based on ^18^F-FDG PET/CT.** PET/CT imaging was collected at baseline and following two weeks of rapamycin treatment. Tumors in FC PIK3ca* mice were identified based on PET avidity and tumor location was confirmed on CT in axial, coronal, and sagittal views (*A*). To estimate tumor volume the area containing the tumor was encircled using Siemens Inveon Research Workplace (*B*). The tumor was then highlighted based on voxel intensity in the region of interest (*C*) and a software generated tumor volume was estimated.
(TIF)

Figure S3 FC PIK3ca* **tumors can be identified by dual hybrid ^18^F-FDG and ^18^F-FLT PET/CT colonography for longitudinal treatment studies.** Untreated FC PIK3ca* mice that were moribund were imaged with ^18^F-FDG (*A*) and ^18^F-FLT (*B*) PET/CT colonography. The PET avid areas correspond to invasive mucinous colonic adenocarcinomas. Three tumors were present in the untreated FC PIK3ca* mouse all three tumors were visualized with PET imaging (*A*). A single proximal colon tumor was visualized with ^8^F-FLT PET/CT colonography in a separate untreated FC PIK3ca* mouse (*B*). Projections are shown on top and axial sections are displayed on the bottom.
(EPS)

Figure S4 **Longitudinal monitoring with dual hybrid ^18^F-FDG PET/CT colonography revealed a dramatic tumor response in** FC PIK3ca* **mice treated with rapamycin.** Further examples of pre- and post-treatment PET imaging demonstrated tumor growth in the placebo-treated mice (*A* and *B*) and tumor response in the rapamycin-treated mice (*C* and *D*).
(EPS)

Figure S5 **pERK1/2 was increased in some untreated** FC PIK3ca* **tumors.** (*A*) A group of ten FC PIK3ca* mice were dissected and the colon and cecal tumors were flash frozen. 5 of 9 proximal colon tumors demonstrated some degree of pERK1/2 signaling. The one cecal tumor identified also demonstrated increased pERK1/2. (*B*) β-catenin staining was performed on multiple rapaymcin and placebo treated tumors. In all instances no nuclear localization of β-catenin was identified, indicating that aberrant WNT signaling is not playing a role in tumorigenesis or resistance to mTOR inhibition. Scale bar: (*B*), 100 µm.
(EPS)

Figure S6 **Rapamycin treatment resulted in decreased pS6 and proliferation in the hyperplastic tissue from the colon of** FC PIK3ca* **mice.** In the colon of FC PIK3ca* mice, hyperplastic tissue was identified (*A*). In the placebo-treated mice, increased pAKT (*B*), pS6 (*C*), and Ki67 (*D*) staining were noted in the hyperplastic epithelium. In the rapamycin-treated mice increased pAKT is observed similar to that in the control, but as expected a decrease in pS6 is seen in response to rapamycin treatment. This reduction in pS6 is associated with a decrease in cell proliferation as determined by Ki67 staining. Size bars: *A–C*, 500 µm; *D*, 200 µm.
(TIF)

Figure S7 **ERK1/2 signaling is able to phosphorylate S6 on Ser^235/236^ through RSK mediated signaling.** ERK1/2 has been demonstrated to activate RSK signaling which results in site-specific phosphorylation of S6 at the Ser^235/236^ site. S6K mediated phosphorylation of S6 can take place at either Ser^235/236^ or Ser^240/244^. (Adapted from Roux, *et al.,* 2007)^24^.
(EPS)

Table S1 **Dual hybrid ^18^F-FDG PET/CT colonography allows for longitudinal monitoring of tumor response and estimation of tumor volumes.**
(DOCX)
