# Supplementary figures and images for: mTOR Inhibition Elicits a Dramatic Response in PI3K-Dependent Colon Cancers
Source: PLoS One. 2013 Apr 9;8(4):e60709. doi: 10.1371/journal.pone.0060709 (PMC3621889; doi:10.1371/journal.pone.0060709)

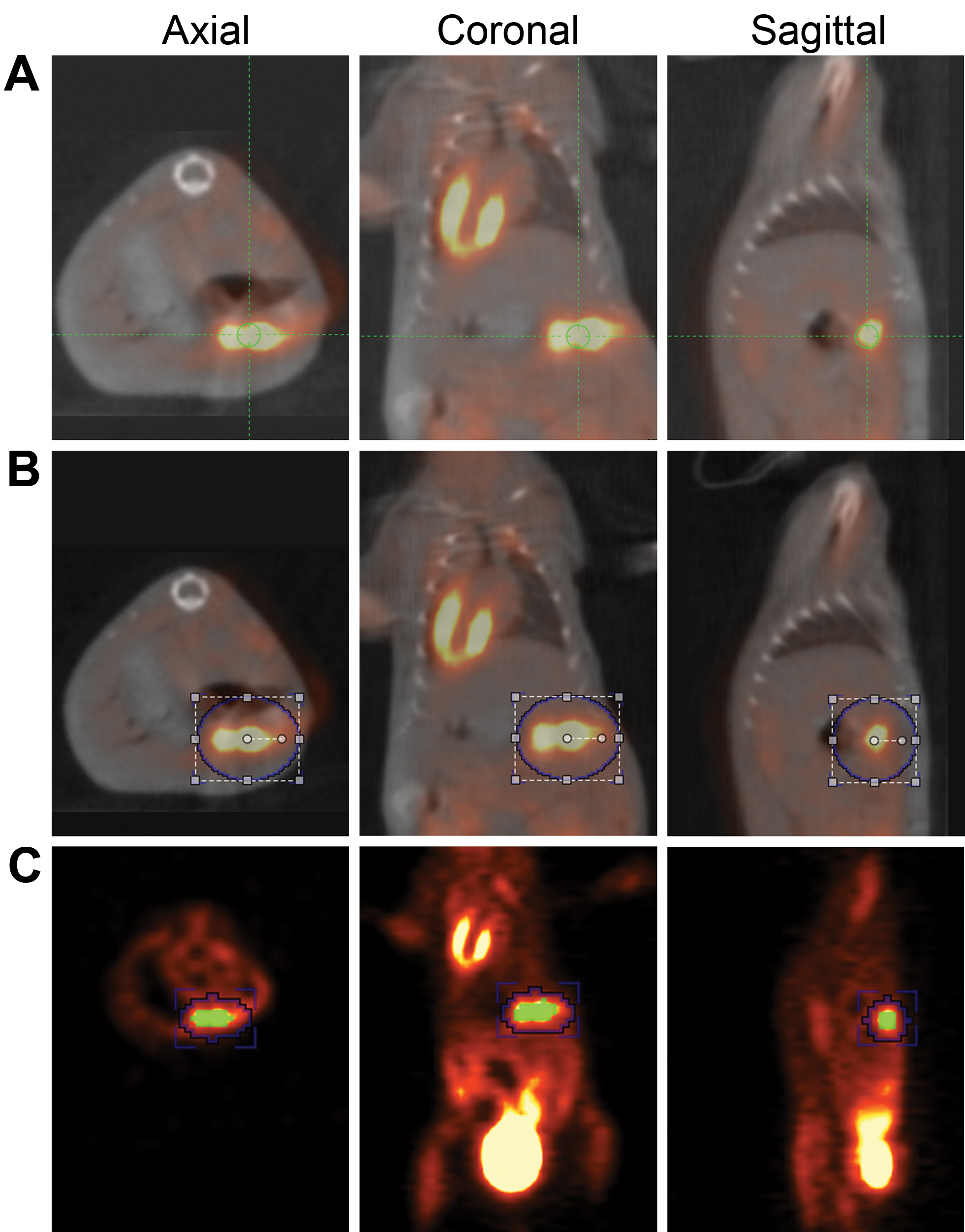

Supplement: Figure S2 — Volume estimation based on 18F-FDG PET/CT. PET/CT imaging was collected at baseline and following two weeks of rapamycin treatment. Tumors in FC PIK3ca* mice were identified based on PET avidity and tumor location was confirmed on CT in axial, coronal, and sagittal views (A). To estimate tumor volume the area containing the tumor was encircled using Siemens Inveon Research Workplace (B). The tumor was then highlighted based on voxel intensity in the region of interest (C) and a software generated tumor volume was estimated. (TIF) [file pone.0060709.s002.tif]

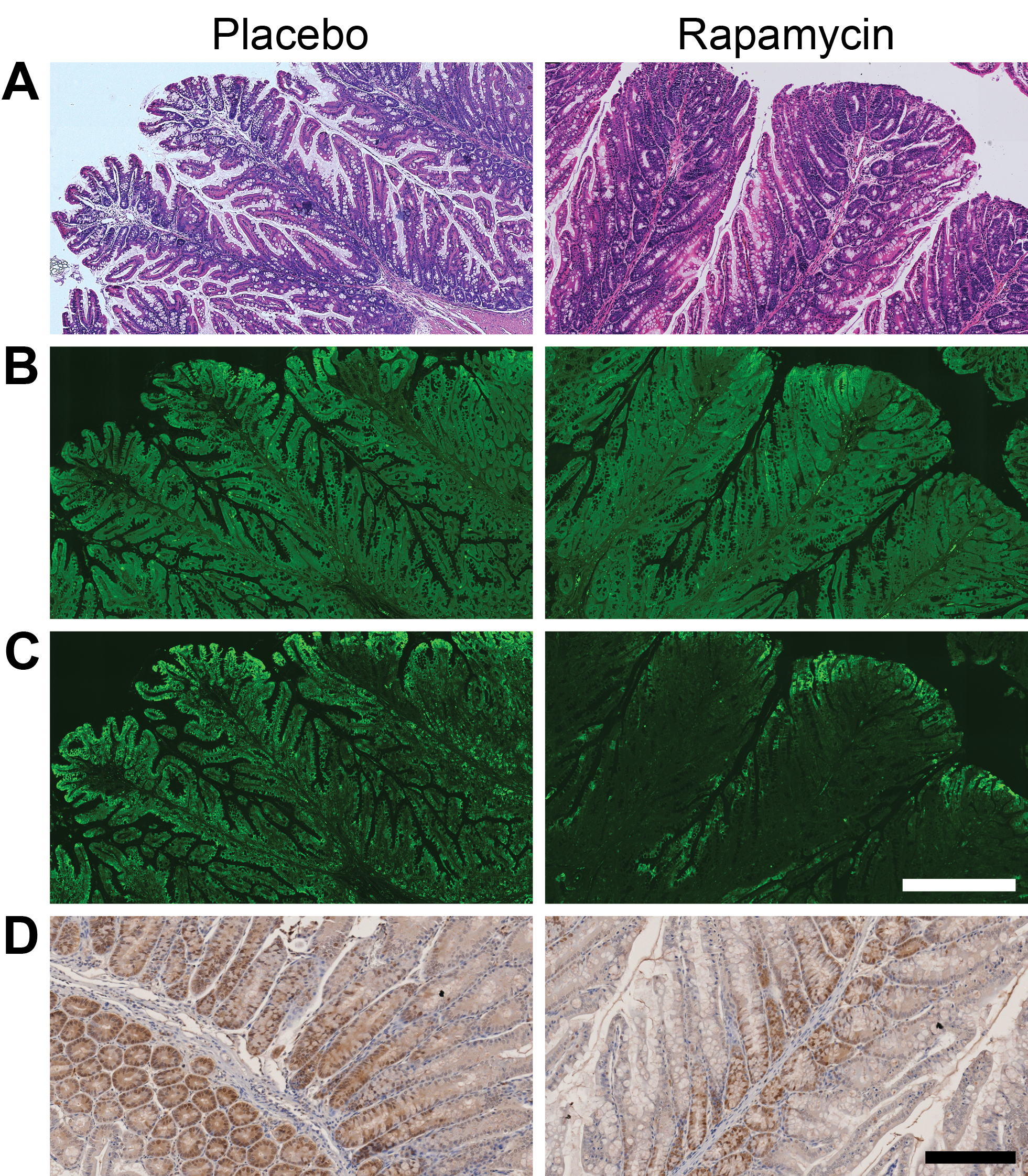

Supplement: Figure S6 — Rapamycin treatment resulted in decreased pS6 and proliferation in the hyperplastic tissue from the colon of FC PIK3ca* mice. In the colon of FC PIK3ca* mice, hyperplastic tissue was identified (A). In the placebo-treated mice, increased pAKT (B), pS6 (C), and Ki67 (D) staining were noted in the hyperplastic epithelium. In the rapamycin-treated mice increased pAKT is observed similar to that in the control, but as expected a decrease in pS6 is seen in response to rapamycin treatment. This reduction in pS6 is associated with a decrease in cell proliferation as determined by Ki67 staining. Size bars: A–C, 500 µm; D, 200 µm. (TIF) [file pone.0060709.s006.tif]
