# Supplementary material for: mTOR Inhibition Elicits a Dramatic Response in PI3K-Dependent Colon Cancers
Source: PLoS One. 2013 Apr 9;8(4):e60709. doi: 10.1371/journal.pone.0060709 (PMC3621889; doi:10.1371/journal.pone.0060709)
Supplement: Table S1 — Dual hybrid 18F-FDG PET/CT colonography allows for longitudinal monitoring of tumor response and estimation of tumor volumes. (DOCX) [file pone.0060709.s008.docx]

**Supplementary Table S1. Dual hybrid ^18^F-FDG PET/CT colonography allows for longitudinal monitoring of tumor response and estimation of tumor volumes.**

|  |  | Tumor volume (mm^3^) | |  |  |
| --- | --- | --- | --- | --- | --- |
| Treatment arm | Mouse# | Baseline | Post-Tx | % Change in volume | Finding on necropsy |
| Placebo | 1 | 51.8 | 162.2 | 213 | Colon tumor |
|  | 2 | 32.6 | 86.8 | 166 | Cecal tumor/metastatic disease |
|  | 3 | 45.1 | 104.1 | 131 | Colon tumor |
|  | 4 | 29.3 | 56.1 | 91 | Colon tumor |
|  | 5 | 41.3 | 74.8 | 81 | No tumor |
|  | 6 | 37.4 | 63.8 | 71 | Colon tumor |
|  | 7 | 23.5 | 36.9 | 57 | Colon tumor |
|  | 8 | 61.9 | 84.9 | 37 | Colon tumor |
|  | 9 | 65.7 | 73.9 | 12 | Cecal tumor |
|  | 21* | 90.2 | NA | NA | Colon tumor |
|  | 22* | 81.6 | NA | NA | Colon tumor |
| Rapamycin | 10 | 86.8 | 55.2 | -36 | Cecal tumor |
|  | 11 | 16.8 | 6.7 | -60 | No tumor |
|  | 12 | 37.4 | 11.5 | -69 | Colon tumor |
|  | 13 | 185.2 | 48.0 | -74 | Colon tumor |
|  | 14 | 114.7 | 28.8 | -75 | Cecal tumor |
|  | 15 | 26.9 | 0 | -100 | No tumor |
|  | 16 | 15.8 | 0 | -100 | No tumor |
|  | 17 | 35.5 | 0 | -100 | No tumor |
|  | 18 | 27.3 | 0 | -100 | Colon tumor |
|  | 19 | 95.0 | 0 | -100 | No tumor |
|  | 20 | 19.7 | 0 | -100 | No tumor |

Twenty-two mice were enrolled and randomized to treatment with placebo or rapamycin. Dual hybrid ^18^F-FDG PET/CT colonography was performed at baseline and following 14 days of treatment. Baseline and post-treatment tumor volume estimates for each mouse are listed. A strong concordance was noted with the PET/CT imaging data and the necropsy results. All of the tumors in the placebo-treated mice grew, while a reduction in tumor volume was noted in all the rapamycin-treated mice. Tx, treatment. *Mice became moribund owing to intestinal obstruction prior to completion of treatment course.
